# Role of IL-17A on Resolution of Pulmonary *C. neoformans* Infection

**DOI:** 10.1371/journal.pone.0017204

**Published:** 2011-02-17

**Authors:** Karen L. Wozniak, Sarah E. Hardison, Jay K. Kolls, Floyd L. Wormley

**Affiliations:** 1 Department of Biology, The University of Texas at San Antonio, San Antonio, Texas, United States of America; 2 The South Texas Center for Emerging Infectious Diseases, The University of Texas at San Antonio, San Antonio, Texas, United States of America; 3 Louisiana State University Health Sciences Center, New Orleans, Louisiana, United States of America; New York University, United States of America

## Abstract

The current studies evaluated the role of interleukin (IL)-17A in the induction of protective immunity against pulmonary cryptococcosis in mice. Protection against pulmonary infection with *C. neoformans* strain H99γ was associated with increased IL-17A production. Signaling through the IFN-γ receptor (R) was required for increased IL-17A production, however, a Th17-type cytokine profile was not observed. Neutrophils were found to be the predominant leukocytic source of IL-17A, rather than T cells, suggesting that the IL-17A produced was not part of a T cell-mediated Th17-type immune response. Depletion of IL-17A in mice during pulmonary infection with *C. neoformans* strain H99γ resulted in an initial increase in pulmonary fungal burden, but had no effect on cryptococcal burden at later time points. Also, depletion of IL-17A did not affect the local production of other cytokines. IL-17RA^−/−^ mice infected with *C. neoformans* strain H99γ survived the primary infection as well as a secondary challenge with wild-type cryptococci. However, dissemination of the wild-type strain to the brain was noted in the surviving IL-17RA^−/−^ mice. Altogether, our results suggested that IL-17A may be important for optimal protective immune responsiveness during pulmonary *C. neoformans* infection, but protective Th1-type immune responses are sufficient for protection against cryptococcal infection.

## Introduction

*Cryptococcus neoformans* is an opportunistic fungal pathogen that causes pneumonia as well as life-threatening meningoencephalitis in individuals with T cell immune deficiencies [1]–[5]. Previous studies have shown that protective immunity against this organism is dependent upon the induction of Th1-type cytokine responses [3], [5]–[16]. Additional studies have also shown that increased IL-17A production is associated with reduced cryptococcal burden [14], [16], [17], suggesting that IL-17A also has a significant role in the generation of protective anti-cryptococcal immune responses.

IL-17A is a proinflammatory cytokine produced by a subset of CD4^+^ T cells, termed Th17 cells (reviewed in [18]–[20]). The primary function of Th17-type T cells is clearance of pathogens that are not adequately handled by Th1 and Th2 cells [20]. Recent reviews suggest that Th17 cells are able to bridge innate and adaptive immune responses [19]. Th17 cells are potent inducers of additional inflammatory mediators, such as TNF-α, IL-1β, and IL-6 (reviewed in [18]). Although the response is termed “Th17” due to the capacity of CD4^+^ T cells to produce IL-17 [18], [19], [21], [22], T cells are not the only source of IL-17. Other sources of IL-17 include γδ T cells, CD8^+^ T cells, NKT cells, NK cells, and neutrophils (reviewed in [19], [20], [23]) [18], [21], [22], [24]–[28]. The cytokines TGF-β and either IL-6 or IL-21 are required to induce IL-17 production from naïve CD4^+^ T cells in mice. IL-21 produced by Th17 cells amplifies the frequency of Th17 cells [20], [29], and IL-23 perpetuates the response and induces IL-17 production from memory CD4^+^ T cells [20]. IL-6 and TGF-β, while inducing Th17 cells, also inhibit the generation of T regulatory (Treg) cells [30]. Furthermore, IL-17A can elicit the production of G-CSF and KC (CXCL1), which both can induce neutrophil chemotaxis [21], [31], [32].

IL-17 has been shown in some infectious disease models, such as *Staphylococcus aureus*, *Bordetella bronchiseptica*, and *Bacteroides fragilis*, to contribute to exacerbated disease [33], [34]. However, in other models of bacterial infection, IL-17 was associated with protective immune responses [33], [35]–[39]. Specifically, Th17 cells have been associated with protective immune responses in the lung against several bacterial pathogens, including *Mycoplasma pneumoniae*, *Bordetella pertussis*, and *Mycobacterium tuberculosis* (reviewed in [26]). Similarly, IL-17 has been shown to have roles in both resistance and susceptibility against a variety of fungal infections. Neutralization of IL-23 or IL-17 during disseminated and oral candidiasis as well as during pulmonary aspergillosis exacerbates pathology, demonstrated by decreased neutrophil infiltration, increased fungal burden, and reduced levels of chemokines [40]–[42]. IL-17 is also associated with protection against *Pneumocystis carinii* infection [43]. In contrast, studies have shown that Th17 cell activation promoted deleterious inflammation and defective fungal clearance in pulmonary aspergillosis and gastrointestinal candidiasis [44]. Further, *in vitro C. neoformans* studies showed that IL-17 treatment reduced yeast proliferation and yeast expulsion from macrophages compared to IL-4 and IL-13 treated macrophages [45]. Previous work in a mouse model of cryptococcal infection suggested that a Th17-type response and IL-17 production are important for modulating survival against cryptococcosis [46]. We have established a model in which infection with an interferon-gamma-producing *C. neoformans* strain, H99γ, elicits protective host immunity against pulmonary cryptococcosis in mice [14], [16]. This model system has been beneficial towards studying protective immunity against pulmonary cryptococcosis. These studies have shown that pulmonary infection with *C. neoformans* strain H99γ, but not wild-type cryptococcci, results in increased pulmonary production of IL-17A [14]. Our observation of high levels of IL-17A in protected mice led to our hypothesis that IL-17A contributes to protective anti-cryptcoccal immune responses. The purpose of these studies was to determine the role of IL-17A in protection against *Cryptococcus neoformans* pulmonary infections.

## Results

### Pulmonary *C. neoformans* strain H99γ infection induces IL-17A production but not a Th17-type cytokine profile

**Figure 1:**
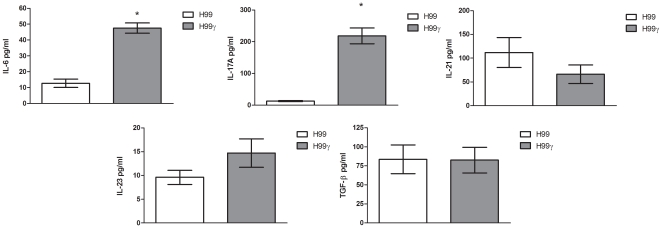
Pulmonary infection with *C. neoformans* strain H99γ results in significant IL-17A, but not Th17, cytokine production in the lungs. BALB/c mice were given an intranasal inoculation with *C. neoformans* strain H99 or H99γ. Lung homogenates were prepared from lungs excised on day 7 post-inoculation and assayed for IL-6, IL-17A, IL-21, IL-23, and TGF-β cytokine production. Data are cumulative of four experiments utilizing 5 mice each per group. Asterisks (*) indicate where significant differences were observed (*P*<0.0001).

Previous studies in our laboratory have shown that infection with an IFN-γ-producing *C. neoformans* strain, H99γ, results in a significant increase in pulmonary IL-17A cytokine production on day 7 post-inoculation compared to mice infected with the parental *C. neoformans* strain H99 [14], [16]. However, it remains unclear whether the increase in IL-17A production observed in the lungs of mice infected with *C. neoformans* strain H99γ is coupled to an overall Th17-type cytokine response. We therefore determined the expression of cytokines associated with the induction of Th17-cytokine responses (IL-6, IL-17A, IL-21, IL-23, and TGF-β) in total lung homogenates derived from mice infected with wild-type *C. neoformans* strain H99 or the transgenic *C. neoformans* strain H99γ on day 7 post-inoculation. We observed a significant increase in IL-6 and IL-17 (Figure 1) in lung homogenates derived from *C. neoformans* strain H99γ infected mice compared to those obtained from mice infected with wild-type yeast (*P*<0.0001 for IL-6 and IL-17A) as previously described [14]. However, no significant differences in the production of IL-21, IL-23, or TGF-β were observed in the lungs of mice infected with wild-type *C. neoformans* strain H99 compared to mice infected with the transgenic *C. neoformans* strain H99γ. Thus, the significant increase in IL-17A production observed in the lungs of mice during infection with the transgenic *C. neoformans* strain H99γ appears not to be associated with a general induction of Th17-type cytokines.

### Neutrophils are the predominant source of IL-17A in mice infected with *C. neoformans* strain H99γ

**Figure 2:**
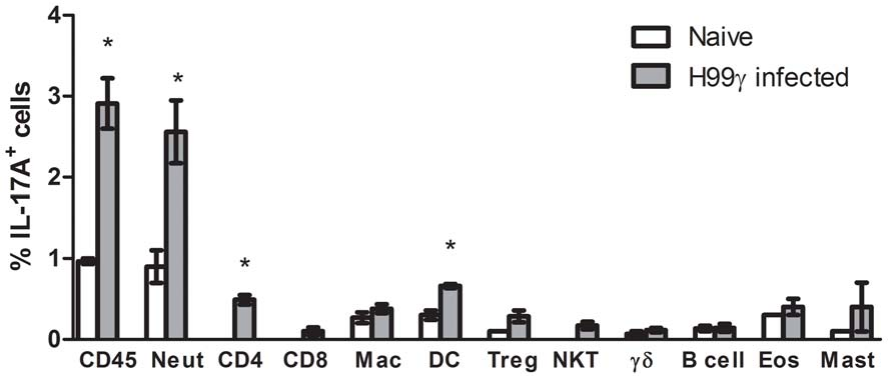
Lung neutrophils are the predominant leukocyte population expressing IL-17A during pulmonary infection with *C. neoformans* strain H99γ. BALB/c mice received an intranasal inoculum of 1×10^4 ^CFU of *C. neoformans* strain H99γ in 50 µl of sterile PBS (gray bars). Naïve Balb/c mice (white bars) are shown as controls. The lungs were excised at day 7 post-inoculation and a single cell suspension generated using enzymatic digestion. The leukocytes were stained with anti-mouse antibodies (CD45, 1A8 (Neut) CD4, CD8, F4/80 (Mac), CD11b/CD11c (DC), CD4/Fox3p (Treg), CD4/DX5 (NKT),γδ, B220 (B cell), SiglecF/CD11b (Eosinophil), FcεR1α/CD117/CD34 (Mast cell)), fixed, permeabilized, and incubated with anti-mouse antibodies specific for IL-17A and quantified by flow cytometry. Flow cytometry data are cumulative results of five independent experiments using pooled leukocytes from 5 mice per group per experiment. Results shown are the percentage of leukocytes expressing the indicated surface markers and IL-17A. Asterisks (*) indicate where significant differences were observed (*P*<0.0001) between naïve mice and mice infected with *C. neoformans* strain H99γ.

We sought to determine the leukocyte population that was the predominant source of IL-17A in the lungs of mice infected with *C. neoformans* strain H99γ. Total leukocytes were isolated from lung tissues of naïve mice and *C. neoformans* strain H99γ-infected mice on day 7 post-challenge, and the lymphocyte subpopulations characterized for intracellular IL-17A expression by flow cytometry. Leukocyte populations examined included CD4^+^ T cells, CD8^+^ T cells, γδ T cells, regulatory T cells, natural killer T cells, macrophages, dendritic cells, neutrophils, B cells, eosinophils, and mast cells. Figure 2 demonstrates that the majority of intracellular IL-17A expression in both naïve mice and mice infected with *C. neoformans* strain H99γ was observed in Ly6G^+^ neutrophils (detected with the 1A8 monoclonal antibody). We also observed that mice infected with *C. neoformans* strain H99γ had significantly higher intracellular IL-17A in total CD45^+^ cells, CD4^+^ T cells, and dendritic cells compared to naïve mice. Although neutrophils appear to be the predominant source of IL-17A, we cannot conclude that increased IL-17A production by other cell types in response to *C. neoformans* strain H99γ infection does not contribute to the observed phenotype. Representative flow cytometry plots are shown in Figure S1.

### Increased pulmonary IL-17A production in *C. neoformans* strain H99γ infected mice involves IFN-γ receptor (R) signaling

**Figure 3:**
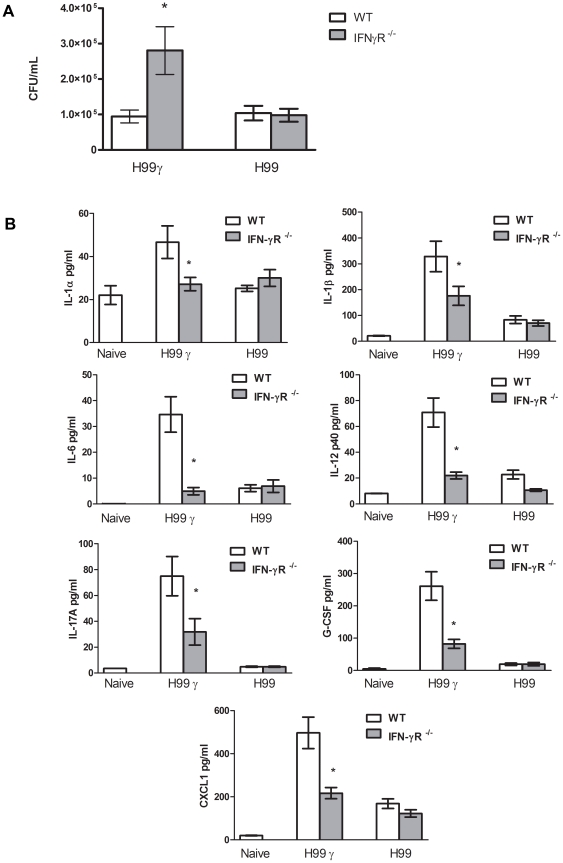
Induction of IL-17A production during *C. neoformans* strain H99γ infection requires IFN-γR expression. BALB/c and IFN-γR^−/−^ mice were given an intranasal inoculation with either *C. neoformans* strain H99 or H99γ. Naïve mice were used as controls for pulmonary cytokines. Lungs were excised at day 7 post-inoculation, and pulmonary cryptococcal burden (A) and cytokine production (B) quantified. Fungal burden results are expressed as mean log CFU per milliliter ± standard errors of the means. A. Asterisks (*) indicate where significant decreases in CFU were observed in IFN-γR ^−/−^ mice compared to wild-type BALB/c mice (*P*<0.01). B. Asterisks (*) indicate where significant differences in cytokines were observed (*P*<0.02). Data are cumulative of two experiments using 5 mice per group.

Our studies suggest that signaling through the IFN-γR may be necessary for the increase in IL-17A production observed in the lungs of mice inoculated with *C. neoformans* strain H99γ. To evaluate this hypothesis, IFN-γR^−/−^ and wild-type (WT) BALB/c mice were inoculated with *C. neoformans* strain H99γ or *C. neoformans* strain H99 and thereafter examined for pulmonary fungal burden, cytokine production, and leukocyte infiltration at day 7 post-infection. Results showed that IFN-γR^−/−^ mice had significantly increased pulmonary fungal burden compared to WT mice when infected with *C. neoformans* strain H99γ, but no change in fungal burden was detected between IFN-γR^−/−^ mice and WT mice during infection with the wild-type strain, *C. neoformans* strain H99 (Figure 3A). Furthermore, cytokine analysis showed that the IFN-γR^−/−^ mice had significantly reduced levels of pro-inflammatory (IL-1α, IL-1β, and IL-6), IL-12p40, IL-17, and chemokine (G-CSF and CXCL1) production compared to WT mice during infection with *C. neoformans* strain H99γ (Figure 3B). No differences were observed in cytokine production in KO vs WT mice infected with *C. neoformans* strain H99.

### Clearance of pulmonary cryptococcosis in IL-17A deficient mice

**Figure 4:**
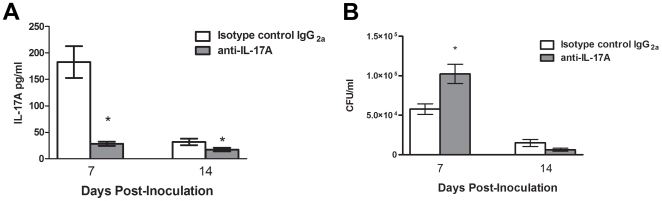
IL-17A depletion does not affect resolution of cryptococcal infection. BALB/c mice were infected with *C. neoformans* strain H99γ, and then treated with 100 µg of anti-IL-17A neutralizing antibody or isotype control IgG_2a_ via the intranasal route four hours following infection. Treatment was repeated every four days thereafter. The lungs from depleted mice and isotype control-treated mice were excised at days 7 and 14 post inoculation and assayed for IL-17A production (A) and cryptococcal fungal burden (B). Data are cumulative of five experiments utilizing 5 mice per group. Asterisks (*) indicate where significant decreases were observed in IL-17A depleted mice compared to isotype-control treated mice following infection with *C. neoformans* strain H99γ (*P*<0.05). Separate mice were used for each time point.

**Figure 5:**
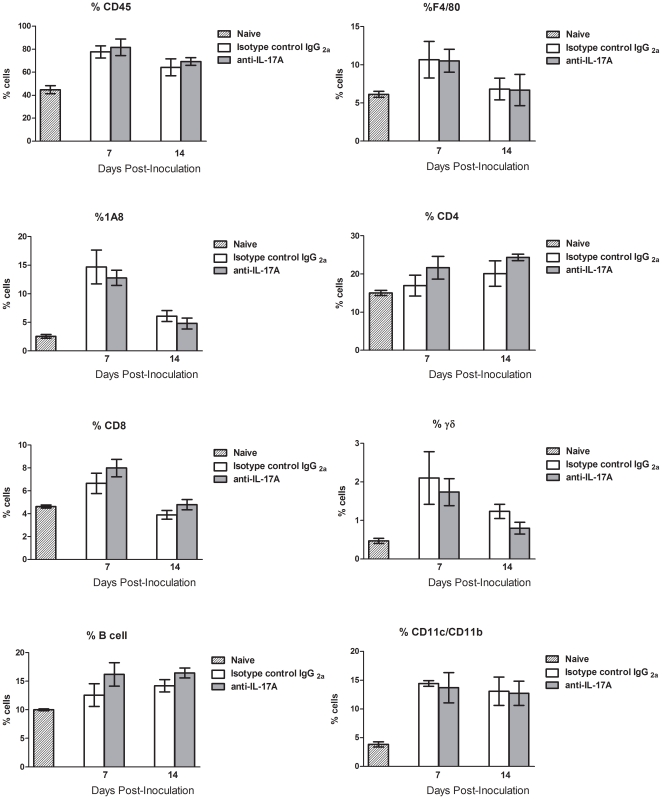
IL-17A depletion does not affect pulmonary leukocyte recruitment. BALB/c mice were infected with *C. neoformans* strain H99γ, and then treated with 100 µg of anti-IL-17A neutralizing antibody or isotype control IgG_2a_ via the intranasal route four hours following infection. Treatment was repeated every four days thereafter. Naïve mice were used as controls for pulmonary leukocyte populations. The lungs from depleted mice and isotype control-treated mice were excised at days 7 and 14 post inoculation and assayed for leukocyte populations by flow cytometry. Data are cumulative of five experiments utilizing 5 mice per group. Separate mice were used for each time point.

Previous studies have suggested that IL-17A may play a role in protection against experimental pulmonary crytococcosis [14]. We therefore examined the effects of IL-17A depletion on the development of protection in mice given an experimental pulmonary infection with *C. neoformans* strain H99γ. Mice were treated intranasally with anti-IL-17A monoclonal antibody or isotype-control antibody beginning at 4 hours post-inoculation and continued every 4 days throughout the experiment. We observed a significant reduction in IL-17A production in lung homogenates of mice treated with the anti-IL-17A antibody at days 7 and 14 post-infection compared to mice treated with the isotype control antibody (Figure 4A). Examination of other cytokines and chemokines in lung homogenates revealed no significant differences between isotype-control and anti-IL-17A antibody treated mice (Table 1). Mice treated with anti-IL-17A antibody had significantly increased pulmonary fungal burden compared to mice treated with the isotype control antibody (*P<*0.002) at day 7 post-infection (Figure 4B). No differences in pulmonary fungal burden were observed at day 14 post-infection (Figure 4B). Although all leukocytes are significantly increased in infected mice compared to naïve controls, we observed no significant differences in leukocyte populations in H99γ-infected isotype control treated mice compared to IL-17A depleted mice at each time point tested (Figure 5).

**Table 1:** Pulmonary cytokine production in IL-17A depleted mice during infection with *C. neoformans* strain H99γ.

	Isotype D7	Anti-IL-17 D7	Isotype D14	Anti-IL-17 D14
**Th1**				
IL-2	29.2±3.7	25.5±3.2	16.3±1.2	13.3±1.1
IL-12p40	118.6±11.3	113.3±12.2	106.5±22.5	111.5±11.1
IL-12p70	29.6±5.2	35.8±5.6	17.5±5.8	17.0±4.5
IFN-γ	22.9±3.5	22.1±4.3	21.3±3.3	19.0±3.8
**Th2**				
IL-4	61.7±8.4	55.1±10.7	12.5±4.8	8.7±8.7
IL-5	7.3±1.1	9.9±2.5	2.7±0.6	2.2±0.4
IL-10	7.8±0.9	7.7±1.2	4.1±0.8	2.9±0.6
**Th17**				
IL-17	182.9± 30.0	28.2±4.0*	31.8±6.2	17.2±3.4*
IL-6	54.2±6.7	58.2±5.5	2.1±0.6	1.2±0.3
**Pro-inflammatory**				
IL-1α	63.2± 6.04	79.9±7.2	35.5± 9.6	27.0±3.0
IL-1β	775.3±102.9	757.0±98.2	455.6±129.1	396.3±81.5
TNF-α	10.2±1.4	9.9±1.4	9.8±2.1	9.4±2.1
G-CSF	375.5±76.7	313.0±41.6	16.0±4.6	10.5±±1.7
**Chemokines**				
MCP-1	1043.0±90.3	1069.0±114.9	203.1±26.4	169.8±9.1
MIP1α	527.4±85.7	562.9±95.8	298.1±41.5	303.1±35.9
MIP1β	69.1±11.8	65.6±12.0	15.2±2.7	11.8±1.3
RANTES	263.4±31.8	280.1±42.0	278.6±36.7	303.3±22.9
KC	584.9±97.6	520.5±92.2	310.0±111.3	175.1±50.9

* =  significant reduction (*P<0.05*) compared to isotype control-treated mice at the same time point.

**Figure 6:**
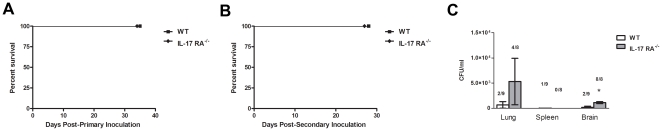
Lack of IL-17R signaling does not significantly affect resolution of infection or survival to cryptococcal infection. BALB/c mice or IL-17RA^−/−^ mice received an intranasal inoculum of *C. neoformans* strain H99γ. Mice were assayed for survival through 35 days of primary infection with *C. neoformans* strain H99γ (A). Surviving mice were then challenged with *C. neoformans* strain H99 and assayed for survival through 28 days of secondary challenge (B). Culture of lung, spleen, and brain tissues at day 28 post-secondary challenge were determined from mice with positive cultures (C) and numbers of mice with positive cultures are shown above each bar. Survival data show one experiment containing nine WT and eight IL-17RA^−/−^ mice. Asterisks (*) indicate where significant differences in fungal burden were observed in IL-17RA^−/−^ mice compared to WT mice following infection with *C. neoformans* strain H99 (*P*<0.0001).

Although antibody depletion significantly reduced the amount of IL-17A in lung homogenates, the depletion was not absolute. Therefore, we evaluated survival of IL-17 receptor A knock-out (IL-17RA^−/−^) mice given a pulmonary infection with *C. neoformans* strain H99γ. IL-17RA^−/−^ mice still have the ability to produce IL-17A, but signaling through its receptor, IL-17RA, is abrogated [47]. Figure 6A demonstrates 100% survival of WT and IL-17RA^−/−^ mice given a primary pulmonary infection with *C. neoformans* strain H99γ through day 35 post-inoculation. All surviving WT and IL-17RA^−/−^mice were subsequently re-challenged with the non-IFN-γ producing wild-type *C. neoformans* strain H99 on day 35 post-primary inoculation and also demonstrated 100% survival through day 28 post-secondary challenge (Figure 5B). Culture of tissue homogenates derived from re-challenged WT and IL-17RA^−/−^ mice at day 28 post-secondary inoculation indicated no significant difference in fungal burden in lung or brain tissues (Figure 6C). Evidence of dissemination to the spleen was not observed in any surviving wild-type mice, but was observed in one surviving knock-out mouse. Interestingly, following differential plating on YPD media with and without nourseothricin (a selectable marker for the H99γ strain), colonization of spleen and brain was due to the challenge organism, strain H99, while the colonization of the lungs was due to the immunizing organism, strain H99γ. Our results suggest that IL-17A contributes to, but is not required for, the resolution of acute infection with *C. neoformans* strain H99γ. However, IL-17RA signaling may be needed to prevent dissemination of cryptococci from the lung to the CNS of immunized mice following re-challenge with WT cryptococci.

## Discussion

IL-17A and the Th17 response have been shown to be important in protection against multiple infectious pathogens. Previous studies suggesting that IL-17A is associated with protective immune responses against *C. neoformans* [16] led us to investigate its contribution in our model of complete protection against experimental pulmonary cryptococcosis. Contrary to reports suggesting that IFN-γ suppresses IL-17A production [39], [48], we found that in the absence of IFN-γR signaling, IL-17A production is significantly reduced during pulmonary infection with *C. neoformans* strain H99γ. Previous results in our laboratory have shown that IL-17A production in IFN-γ KO mice is similar to that observed in WT mice following infection with *C. neoformans* strain H99γ [49]. Therefore, signaling through the IFN-γ receptor may directly or indirectly influence IL-17A production in our model system, but is not entirely responsible for IL-17A production. Further, the presence or absence of the IFN-γ cytokine does not affect IL-17A production [49]. These results suggest that in our model, IL-17A works in collaboration with the protective Th1-type immune response as reported in studies of protection against other infectious pathogens [18], [45].

Elevated IL-17A production in the lungs correlates with protective immune responses against cryptococcal infection and resolution of infection [14], [16]. Indeed, our results herein suggest that IL-17A may be involved in the protective response at early time points, since increased fungal burden is evident in mice treated with anti-IL-17A at day 7 post-inoculation with *C. neoformans* strain H99γ. One important function of IL-17A, that of a neutrophil chemoattractant [21], [31], was not observed in our studies (Figure 5). Also, depletion of IL-17A during infection with *C. neoformans* strain H99γ resulted in no changes in any other pulmonary cytokine tested. Despite early differences in pulmonary fungal burden, mice depleted of IL-17A and IL-17RA^−/−^ mice eventually resolve the pulmonary infection, suggesting that alternative protective mechanisms may be present in mice lacking IL-17A or in mice lacking signalling through the IL-17 receptor. Interestingly, the surviving IL-17R^−/−^ animals all had detectable brain colonization with *C. neoformans* strain H99, suggesting that these animals had difficulty preventing dissemination of the wild-type cryptococci, even though they showed no signs or symptoms of disease.

Our studies examining IL-17A expression within lung leukocytes during *C. neoformans* strain H99γ infection suggest that neutrophils are the predominant source of IL-17A, rather than CD4^+^ T cells, which has been shown in other systems [22], [50], [51]. Results from other fungal infection model systems also suggested that multiple cell types can produce IL-17A (reviewed in [28]). Our data suggest that the IL-17A produced during protective anti-*C. neoformans* immune responses is not associated with an overall Th17-type cytokine response. Th17 responses are associated with the production of IL-21, IL-23, and TGF-β; none of which are significantly increased in the lungs of protected mice in our model (Figure 1). IL-17RA deficiency as well as treatment with anti-IL-17A neutralizing antibodies has been shown to decrease neutrophil infiltration and production of G-CSF [23]. Further, IL-17A regulates neutrophils by inducing G-CSF production and controls expansion of IL-17A-producing neturophil regulating T cells through the IL-17RA [23]. In contrast, we did not observe any change in neutrophil recruitment in mice treated with anti-IL-17A antibodies (Figure 5), further suggesting that IL-17A alone was not required to induce neutrophil recruitment to the lungs in response to *C. neoformans* H99γ infection.

Since complete protection is only observed during acute infection in mice infected with *C. neoformans* strain H99γ and not in mice infected with wild-type cryptococci, this model has been beneficial in evaluating the impact of various components of host immunity on the resolution of experimental pulmonary cryptococcosis. In our model, infection of mice with an IFN-γ-producing *C. neoformans* strain, H99γ, leads to increased production of IL-17A, resolution of the acute infection, and protection against challenge with wild-type *C. neoformans* [14], [16], [52]. Our studies show that the significant increase in IL-17A production requires IFN-γ receptor signaling and is likely not associated with a Th17-type immune response. Furthermore, neutrophils appeared to be the predominant leukocyte population expressing IL-17A. Taken together, our studies show that although IL-17A may contribute to immune defenses against pulmonary infection with *C. neoformans* strain H99γ, alternative mechanisms, namely Th1-type host responses, are predominantly responsible for the resolution of pulmonary *C. neoformans* infection.

## Materials and Methods

### Ethics

This study was carried out in strict accordance with the recommendations in the Guide for the Care and Use of Laboratory Animals of the National Institutes of Health. Mice were housed at The University of Texas at San Antonio Small Animal Laboratory Vivarium. These animal experiments were approved by The University of Texas at San Antonio Institutional Animal Care and Use Committee (IACUC), approved protocol number MU021-11/11A2, and mice were handled according to IACUC guidelines. All efforts were made to minimize animal suffering.

### Mice

Female BALB/c (H-2^d^) (National Cancer Institute/Charles River Laboratories, Boston, MA and The Jackson Laboratory, Bar Harbor, ME), IFN-γR^−/−^ (The Jackson Laboratory), and IL-17 receptor A knock out (IL-17RA^−/−^) mice (a kind gift of Jay K. Kolls, Louisiana State University Health Sciences Center, New Orleans, LA), all on the BALB/c background with an average weight of 20–25 grams, were used throughout these studies.

### Strains and media

*C. neoformans* strains H99 (serotype A, Mat α) and H99γ (serotype A, Mat α, an interferon-gamma producing strain derived from *C. neoformans* H99 [14]) were recovered from 15% glycerol stocks stored at –80°C prior to use in the experiments described herein. The strains were maintained on yeast-extract-peptone-dextrose (YPD) media (1% yeast extract, 2% peptone, 2% dextrose, and 2% Bacto agar). Yeast cells were grown for 18–20 h at 30°C with shaking in YPD broth (Becton Dickinson and Company, Sparks, MD), collected by centrifugation, washed three times with sterile phosphate-buffered saline (PBS), and viable yeast quantified using trypan blue dye exclusion in a hemacytometer.

### Pulmonary infections

Pulmonary *C. neoformans* infections were initiated by nasal inhalation as previously described [16], [53], [54]. BALB/c mice were anesthetized with 2% isoflurane using a rodent anesthesia device (Eagle Eye Anesthesia, Jacksonville, FL) and then given a yeast inoculum of 1×10^4^ colony forming units (CFU) of *C. neoformans* strains H99 or H99γ in 50 µl of sterile PBS pipetted directly into the nares. The inocula used were verified by quantitative culture on YPD agar. The mice were fed ad libitum and were monitored by inspection twice daily. Mice were euthanized at specific time points post-inoculation by CO_2_ inhalation followed by cervical dislocation, and lung tissues were excised using aseptic technique. Tissues were homogenized in 1 ml of sterile PBS, followed by culture of 10-fold dilutions of each tissue on YPD agar supplemented with chloramphenicol (Mediatech, Inc., Herndon, VA). CFU were enumerated following incubation at 30°C for 48 h. Alternatively, mice intended for survival analysis were monitored by inspection twice daily and euthanized if they appeared to be in pain or moribund.

### Pulmonary leukocyte isolation

Lungs were excised at specific time points post- inoculation and digested enzymatically at 37°C for 30 minutes in 10 ml of digestion buffer (RPMI 1640 and 1 mg/ml of collagenase type IV [Sigma-Aldrich, St. Louis, MO.]) with intermittent (every 10 min) stomacher homogenizations. The enzymatically-digested tissues were then successively filtered through sterile nylon filters of various pore sizes (70 and 40 µm) (BD Biosciences) and washed with sterile HBSS to enrich for leukocytes. Erythrocytes were lysed by incubation in NH_4_Cl buffer (0.859% NH_4_Cl, 0.1% KHCO_3_, 0.0372% Na_2_EDTA [pH 7.4]; Sigma-Aldrich) for 3 minutes on ice followed by the addition of a 10-fold excess of PBS. The resulting leukocyte population was then collected by centrifugation (800×*g*) for 5 minutes, washed twice with sterile PBS, resuspended in sterile PBS+ 2% heat-inactivated fetal bovine serum (FACS buffer) and enumerated in a hemacytometer using trypan blue dye exclusion. Flow cytometric analysis was used to determine the percentage of each leukocyte population as well as the absolute number of total leukocytes (CD45^+^) within the lung cell suspension for standardization of hemacytometer counts.

### Cytokine depletions

For anti-IL-17A experiments, mice received 100 µg anti-IL-17A in a volume of 25 µl pipetted directly into the nares, beginning 4 hours post-inoculation and continuing every 4 days throughout the study. Controls for these experiments included mice treated with isotype control antibody via the intranasal route.

### Antibodies

For flow cytometry experiments, cells were incubated with CD16/CD32 (Fc Block™) (BD Biosciences, San Diego, CA) and the following antibodies conjugated to phycoerythrin (PE), allophycocyanin (APC), Alexa 647, or PECy7 were added: a cocktail of CD3, CD4, and CD8α; CD45, MHC class II, B220, Siglec-F (BD Biosciences), 1A8, CD11c, CD11b, F4/80, DX5, Fox3P, γδ, MHC class II, CD86, IL-17A, FcεR1α CD117, CD34 (eBioscience Inc.), and F4/80 (Caltag Laoratories, Burlingame, CA).

### Flow cytometry

Standard methodology was employed for the direct immunofluorescence of pulmonary leukocytes. Briefly, in 96-well U-bottom plates, 100 µl containing 1×10^6^ cells in PBS + 2% FBS (FACS buffer) were incubated with 50 µl of Fc Block™ (BD Biosciences) diluted in FACS buffer for 5 minutes to block non-specific binding of antibodies to cellular Fc receptors. Subsequently, an optimal concentration of fluorochrome-conjugated antibodies (between 0.06–0.5 µg/1×10^6^ cells in 50 µl of FACS buffer) were added in various combinations to allow for dual or triple staining experiments, and plates were incubated for 30 minutes at 4°C. Following incubation, the cells were washed three times with FACS buffer and cells were fixed in 200 µl of 2% ultrapure formaldehyde (Polysciences, Inc., Warrington, PA) diluted in FACS buffer (fixation buffer). For intracellular staining, cells remained in fixation buffer for 10 min at room temperature. After fixation, the cells were washed and permeabilized with 0.1% saponin for 10 min at room temperature. While permeabilized, the cells were intracellularly stained with anti-IL-17A (eBioscience Inc.) and/or anti-Fox3P (regulatory T cell) (eBioscience Inc.) for 30 min at 4°C. Cells were then washed 3 times with 0.1% saponin and then resuspended in fixation buffer before flow cytometry was performed. Cells incubated with either FACS buffer alone or single fluorochrome-conjugated antibodies were used to determine positive staining and spillover/compensation calculations, and the flow cytometer determined background fluorescence. The samples were analyzed using BD FACSArray software™ on a BD FACSArray flow cytometer (BD Biosciences). Dead cells were excluded on the basis of forward angle and 90° light scatter. For data analyses, 30,000 events (cells) were evaluated from a predominantly leukocytic population identified by backgating from CD45^+^-stained cells. The absolute number of total leukocytes was quantified by multiplying the total number of cells observed by hemacytometer counting by the percentage of CD45^+^ cells determined by flow cytometry. The absolute number of each leukocyte subset (1A8, F4/80^+^, CD11c^+^/CD11b^+^, CD11c^+^/CD11b^+^/MHC class II^+^, CD11c^+^/CD11b^+^/CD86^+^, B220^+^, MHC class II^+^, CD4^+^/CD3^+^ CD8^+^/CD3^+^, CD4^+^/Fox3p^+^, CD4^+^/DX5^+^, Siglec-F^+^/CD11b^+^, FcεR1α^+^/CD117^+^/CD34^+^ was determined by multiplying the percentage of each gated population by the total number of CD45^+^ cells.

### Cytokine analysis

Cytokine levels in lung tissues were analyzed using the Bio-Plex Protein Array System (Luminex-based technology) (Bio-Rad Laboratories, Hercules, CA). Briefly, lung tissue was excised and homogenized in ice-cold sterile PBS (1 ml). An aliquot (50 µl) was taken to quantify the pulmonary fungal burden and an anti-protease buffer solution (1 ml) containing PBS, protease inhibitors (inhibiting cysteine, serine, and other metalloproteinases) and 0.05% Triton X-100 was added to the homogenate. Samples were then clarified by centrifugation (800×g) for 5 minutes. Supernatants from pulmonary homogenates were assayed using the Bio-Plex Protein Array System (Bio-Rad Laboratories) for the presence of interferon (IFN)- γ, interleukin (IL)-1α, IL-1β, IL-2, IL-4, IL-5, IL-10, IL-12 p70, IL-17, tumor necrosis factor (TNF)- α, and granulocyte-colony stimulating factor [G-CSF] expression as well as chemokines (macrophage inflammatory protein [MIP]-1α (CCL3), MIP-1β (CCL4), macrophage chemoattractant protein [MCP]-1 (CCL2), keratinocyte-derived chemokine (KC) (CXCL1), and regulated upon activation, normal T cell expressed and secreted [RANTES] (CCL5)). ELISA assays were performed to measure TGF-β, IL-23 (R&D Systems), and IL-21 (BD Biosciences) on pulmonary homogenates.

### Statistical analysis

The unpaired Student's *t* test (two-tailed) was used to analyze fungal burden, pulmonary cell populations, and cytokine/chemokine data using GraphPad Prism version 5.00 for Windows (GraphPad Prism Software, San Diego California USA). Survival data was analyzed using the log-rank test (GraphPad Prism Software). Significant differences were defined as *P*<0.05.

## Supporting Information

Figure S1 **Lung neutrophils are the predominant leukocyte population expression IL-17A during pulmonary infection with** ***C. neoformans*** **strain H99γ.** BALB/c mice received an intranasal inoculum of 1×10^4 ^CFU of *C. neoformans* strain H99γ in 50 µl of sterile PBS. Naïve Balb/c mice are shown as controls. The lungs were excised at day 7 post-inoculation and a single cell suspension generated using enzymatic digestion. The leukocytes were stained with anti-mouse antibodies (CD45, 1A8 (Neut) CD4, CD8, F4/80 (Mac), CD11b/CD11c (DC), CD4/Fox3p (Treg), CD4/DX5 (NKT),γδ, B220 (B cell), SiglecF/CD11b (Eosinophil), FcεR1α/CD117/CD34 (Mast cell)), fixed, permeabilized, and incubated with anti-mouse antibodies specific for IL-17A and quantified by flow cytometry. Flow cytometry dot plots are representative data of five independent experiments using pooled leukocytes from 5 mice per group per experiment. Results shown in the upper right quadrant of each plot are the percentage of leukocytes expressing the indicated surface markers and IL-17A.
(TIF)

## References

[1] Levitz SM (1991). The ecology of *Cryptococcus neoformans* and the epidemiology of cryptococcosis.. Rev Infect Dis.

[2] Mitchell TG, Perfect JR (1995). Cryptococcosis in the Era of AIDS - 100 years after the discovery of *Cryptococcus neoformans*.. Clin Microbiol Rev.

[3] Shoham S, Levitz SM (2005). The immune response to fungal infections.. Br J Haematol.

[4] Singh N, Gayowski T, Wagener MM, Marino IR (1997). Clinical spectrum of invasive cryptococcosis in liver transplant recipients receiving tacrolimus.. Clin Transplant.

[5] Singh N, Dromer F, Perfect JR, Lortholary O (2008). Cryptococcosis in solid organ transplant recipients: current state of the science.. Clin Infect Dis.

[6] Hill JO, Harmsen AG (1991). Intrapulmonary growth and dissemination of an avirulent strain of *Cryptococcus neoformans* in mice depleted of CD4^+^ or CD8^+^ T-Cells.. J Exp Med.

[7] Huffnagle GB, Lipscomb MF, Lovchik JA, Hoag KA, Street NE (1994). The role of CD4(+) and CD8(+) T-Cells in the protective inflammatory response to a pulmonary cryptococcal infection.. J Leukoc Biol.

[8] Huffnagle GB, Yates JL, Lipscomb MF (1991). Immunity to a pulmonary *Cryptococcus neoformans* infection requires both CD4^+^ and CD8^+^ T-Cells.. J Exp Med.

[9] Huffnagle GB, Yates JL, Lipscomb MF (1991). T-cell-mediated immunity in the lung - a *Cryptococcus neoformans* pulmonary infection model using SCID and athymic nude-mice.. Infect Immun.

[10] Mody CH, Lipscomb MF, Street NE, Toews GB (1990). Depletion of CD4+ (L3T4+) lymphocytes in vivo impairs murine host defense to *Cryptococcus neoformans*.. J Immunol.

[11] Chuck SL, Sande MA (1989). Infections with *Cryptococcus neoformans* in the Acquired Immunodeficiency Syndrome.. New Engl J Med.

[12] Blasi E, Mazzolla R, Barluzzi R, Mosci P, Bistoni F (1994). Anticryptococcal resistance in the mouse brain: beneficial effects of local administration of heat-inactivated yeast cells.. Infect Immun.

[13] Buchanan KL, Doyle HA (2000). Requirement for CD4^+^ T Lymphocytes in Host Resistance against *Cryptococcus neoformans* in the Central Nervous System of Immunized Mice.. Infect Immun.

[14] Wormley FL, Perfect JR, Steele C, Cox GM (2007). Protection Against Cryptococcosis using a Murine Interferon-gamma Producing *Cryptococcus neoformans* Strain.. Infect Immun.

[15] Young M, Macias S, Thomas D, Wormley FL (2009). A proteomic-based approach for the identification of immunodominant *Cryptococcus neoformans* proteins.. Proteomics.

[16] Wozniak KL, Ravi S, Macias S, Young ML, Olszewski MA (2009). Insights into the mechanisms of protective immunity against *Cryptococcus neoformans* infection using a mouse model of pulmonary cryptococcosis.. PLoS ONE.

[17] Kleinschek MA, Muller U, Brodie SJ, Stenzel W, Kohler G (2006). IL-23 Enhances the Inflammatory Cell Response in *Cryptococcus neoformans* Infection and Induces a Cytokine Pattern Distinct from IL-12.. J Immunol.

[18] Mills KH (2008). Induction, function and regulation of IL-17-producing T cells.. Eur J Immunol.

[19] Peck A, Mellins ED (2010). Precarious balance: Th17 cells in host defense.. Infect Immun.

[20] Korn T, Bettelli E, Oukka M, Kuchroo VK (2009). IL-17 and Th17 Cells.. Annu Rev Immunol.

[21] Ley K, Smith E, Stark MA (2006). IL-17A-producing neutrophil-regulatory Tn lymphocytes.. Immunol Res.

[22] Ferretti S, Bonneau O, Dubois GR, Jones CE, Trifilieff A (2003). IL-17, produced by lymphocytes and neutrophils, is necessary for lipopolysaccharide-induced airway neutrophilia: IL-15 as a possible trigger.. J Immunol.

[23] Smith E, Stark MA, Zarbock A, Burcin TL, Bruce AC (2008). IL-17A inhibits the expansion of IL-17A-producing T cells in mice through “short-loop” inhibition via IL-17 receptor.. J Immunol.

[24] Song C, Luo L, Lei Z, Li B, Liang Z (2008). IL-17-producing alveolar macrophages mediate allergic lung inflammation related to asthma.. J Immunol.

[25] Kish DD, Li X, Fairchild RL (2009). CD8 T cells producing IL-17 and IFN-gamma initiate the innate immune response required for responses to antigen skin challenge.. J Immunol.

[26] Nembrini C, Marsland BJ, Kopf M (2009). IL-17-producing T cells in lung immunity and inflammation.. J Allergy Clin Immunol.

[27] Braun RK, Ferrick C, Neubauer P, Sjoding M, Sterner-Kock A (2008). IL-17 producing gammadelta T cells are required for a controlled inflammatory response after bleomycin-induced lung injury.. Inflammation.

[28] Romani L, Zelante T, De Luca A, Fallarino F, Puccetti P (2008). IL-17 and therapeutic kynurenines in pathogenic inflammation to fungi.. J Immunol.

[29] Korn T, Bettelli E, Gao W, Awasthi A, Jager A (2007). IL-21 initiates an alternative pathway to induce proinflammatory T(H)17 cells.. Nature.

[30] Bettelli E, Carrier Y, Gao W, Korn T, Strom TB (2006). Reciprocal developmental pathways for the generation of pathogenic effector TH17 and regulatory T cells.. Nature.

[31] Linden A, Laan M, Anderson GP (2005). Neutrophils, interleukin-17A and lung disease.. Eur Respir J.

[32] Witowski J, Pawlaczyk K, Breborowicz A, Scheuren A, Kuzlan-Pawlaczyk M (2000). IL-17 Stimulates Intraperitoneal Neutrophil Infiltration Through the Release of GRO{alpha} Chemokine from Mesothelial Cells.. J Immunol.

[33] Chung DR, Kasper DL, Panzo RJ, Chtinis T, Grusby MJ (2003). CD4+ T Cells Mediate Abscess Formation in Intra-abdominal Sepsis by an IL-17-Dependent Mechanism.. J Immunol.

[34] Siciliano NA, Skinner JA, Yuk MH (2006). *Bordetella bronchiseptica* modulates macrophage phenotype leading to the inhibition of CD4+ T cell proliferation and the initiation of a Th17 immune response.. J Immunol.

[35] Freitas A, Alves-Filho JC, Victoni T, Secher T, Lemos HP (2009). IL-17 receptor signaling is required to control polymicrobial sepsis.. J Immunol.

[36] Miyamoto M, Prause O, Sjostrand M, Laan M, Lotvall J (2003). Endogenous IL-17 as a mediator of neutrophil recruitment caused by endotoxin exposure in mouse airways.. J Immunol.

[37] Ye P, Garvey PB, Zhang P, Nelson S, Bagby G (2001). Interleukin-17 and lung host defense against *Klebsiella pneumoniae* infection.. Am J Respir Cell Mol Biol.

[38] Happel KI, Zheng M, Young E, Quinton LJ, Lockhart E (2003). Cutting edge: roles of Toll-like receptor 4 and IL-23 in IL-17 expression in response to *Klebsiella pneumoniae* infection.. J Immunol.

[39] McKenzie BS, Kastelein RA, Cua DJ (2006). Understanding the IL-23-IL-17 immune pathway.. Trends Immunol.

[40] Conti HR, Shen F, Nayyar N, Stocum E, Sun JN (2009). Th17 cells and IL-17 receptor signaling are essential for mucosal host defense against oral candidiasis.. J Exp Med.

[41] Huang W, Na L, Fidel PL, Schwarzenberger P (2004). Requirement of interleukin-17A for systemic anti-*Candida albicans* host defense in mice.. J Infect Dis.

[42] Werner JL, Metz AE, Horn D, Schoeb TR, Hewitt MM (2009). Requisite role for the dectin-1 beta-glucan receptor in pulmonary defense against *Aspergillus fumigatus*.. J Immunol.

[43] Rudner XL, Happel KI, Young EA, Shellito JE (2007). Interleukin-23 (IL-23)-IL-17 cytokine axis in murine *Pneumocystis carinii* infection.. Infect Immun.

[44] Zelante T, De Luca A, Bonifazi P, Montagnoli C, Bozza S (2007). IL-23 and the Th17 pathway promote inflammation and impair antifungal immune resistance.. Eur J Immunol.

[45] Voelz K, Lammas DA, May RC (2009). Cytokine signaling regulates the outcome of intracellular macrophage parasitism by *Cryptococcus neoformans*.. Infect Immun.

[46] Muller U, Stenzel W, Kohler G, Werner C, Polte T (2007). IL-13 Induces Disease-Promoting Type 2 Cytokines, Alternatively Activated Macrophages and Allergic Inflammation during Pulmonary Infection of Mice with *Cryptococcus neoformans*.. J Immunol.

[47] Ye P, Rodriguez FH, Kanaly S, Stocking KL, Schurr J (2001). Requirement of interleukin 17 receptor signaling for lung CXC chemokine and granulocyte colony-stimulating factor expression, neutrophil recruitment, and host defense.. J Exp Med.

[48] Lee YK, Turner H, Maynard CL, Oliver JR, Chen D (2009). Late developmental plasticity in the T helper 17 lineage.. Immunity.

[49] Hardison SE, Wozniak KL, Kolls JK, Wormley FL (2010). Interleukin-17 Is Not Required for Classical Macrophage Activation in a Pulmonary Mouse Model of *Cryptococcus neoformans* Infection.. Infect Immun.

[50] Romani L, Fallarino F, De Luca A, Montagnoli C, D'Angelo C (2008). Defective tryptophan catabolism underlies inflammation in mouse chronic granulomatous disease.. Nature.

[51] Andreasen C, Powell DA, Carbonetti NH (2009). Pertussis toxin stimulates IL-17 production in response to *Bordetella pertussis* infection in mice.. PLoS ONE.

[52] Hardison SE, Ravi S, Wozniak KL, Young ML, Olszewski MA (2010). Pulmonary infection with an interferon-gamma-producing *Cryptococcus neoformans* strain results in classical macrophage activation and protection.. Am J Pathol.

[53] Cox GM, McDade HC, Chen SC, Tucker SC, Gottfredsson M (2001). Extracellular phospholipase activity is a virulence factor for *Cryptococcus neoformans*.. Mol Microbiol.

[54] Cox GM, Mukherjee J, Cole GT, Casadevall A, Perfect JR (2000). Urease as a virulence factor in experimental cryptococcosis.. Infect Immun.

