# Supplementary figures and images for: Role of IL-17A on Resolution of Pulmonary C. neoformans Infection
Source: PLoS One. 2011 Feb 17;6(2):e17204. doi: 10.1371/journal.pone.0017204 (PMC3040760; doi:10.1371/journal.pone.0017204)

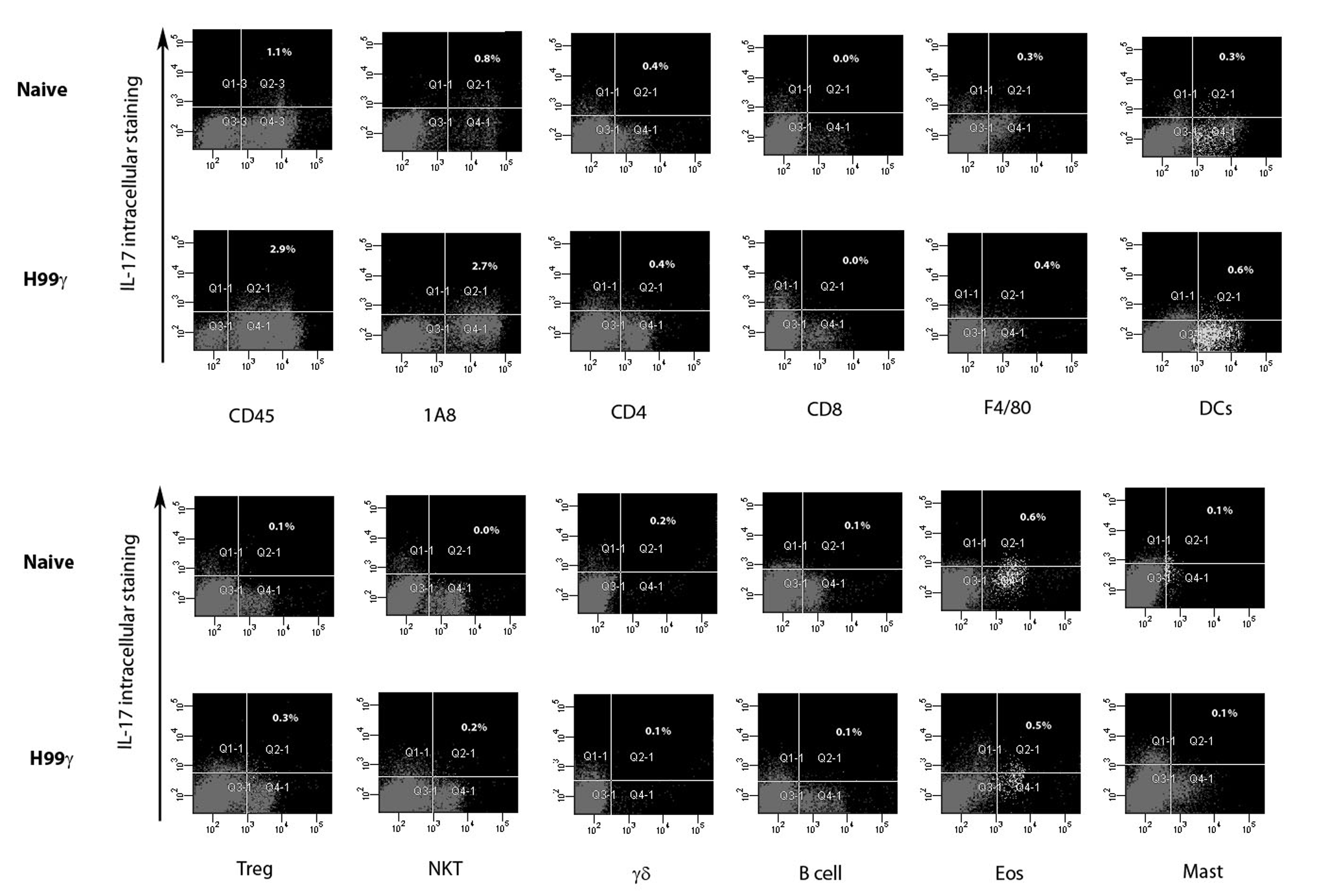

Supplement: Figure S1 — Lung neutrophils are the predominant leukocyte population expression IL-17A during pulmonary infection with C. neoformans strain H99γ. BALB/c mice received an intranasal inoculum of 1×10^4^ CFU of C. neoformans strain H99γ in 50 µl of sterile PBS. Naïve Balb/c mice are shown as controls. The lungs were excised at day 7 post-inoculation and a single cell suspension generated using enzymatic digestion. The leukocytes were stained with anti-mouse antibodies (CD45, 1A8 (Neut) CD4, CD8, F4/80 (Mac), CD11b/CD11c (DC), CD4/Fox3p (Treg), CD4/DX5 (NKT),γδ, B220 (B cell), SiglecF/CD11b (Eosinophil), FcεR1α/CD117/CD34 (Mast cell)), fixed, permeabilized, and incubated with anti-mouse antibodies specific for IL-17A and quantified by flow cytometry. Flow cytometry dot plots are representative data of five independent experiments using pooled leukocytes from 5 mice per group per experiment. Results shown in the upper right quadrant of each plot are the percentage of leukocytes expressing the indicated surface markers and IL-17A. (TIF) [file pone.0017204.s001.tif]
